# Geophysics for the environment in Indonesia

**DOI:** 10.12688/f1000research.145869.1

**Published:** 2024-02-22

**Authors:** Achmad Darul, Dasapta Erwin Irawan, Eleonora Agustine

**Affiliations:** 1 Departement of Geology, Faculty of Industrial Engineering, Institut Teknologi Sumatera, Way Hui, South Lampung, Indonesia; 2 Applied Geology Research Group, Faculty of Earth Sciences and Technology, Institut Teknologi Bandung, Bandung, West Java, Indonesia; 3 Faculty of Geology, Universitas Padjadjaran, Bandung, West Java, Indonesia

**Keywords:** geophysics, environmental problems, urban areas, urban hydrogeology, subsurface analyses, geology, aquifers, groundwater

## Abstract

This paper explores the hidden potential of geophysics for the environment, focusing on subsurface mapping activities in Indonesia. Geophysics plays a crucial role in understanding the Earth’s physical characteristics and addressing environmental challenges. It is particularly relevant in water-related environmental problems, such as groundwater contamination and infiltration monitoring. Geophysics is also used to detect metals in fertile soils and plants, providing insights into agricultural practices and potential health risks. However, applying geophysics in urban areas poses challenges due to physical obstructions, cultural noise, limited workspace, permits, and safety concerns.

This article emphasizes the integration of geophysics with environmental studies, the need for further research on water-related environmental problems and metal detection, and the development of techniques tailored for urban environments. It suggests focusing on understanding the specific environmental challenges in Indonesia and leveraging advancements in technology for more accurate and efficient geophysical investigations.

In the Indonesian context, geophysics has diverse applications, including energy exploration, seismology, and oceanography. However, it has not been properly utilized in the field of environmental studies, particularly in urban areas.

## Introduction

Geophysics plays a crucial role in environmental studies by providing valuable insights into the Earth’s physical characteristics and the living environment of mankind. Environmental geophysics, also known as near-surface geophysics, integrates environmental science and geophysics to study the relationship between geophysical fields and the Earth’s physical characteristics, including natural and artificial environments. ^1^ Geophysical techniques are pivotal tools for detecting and studying various environmental problems caused by drinking and wastewater, as well as delineating contaminated, partly affected, and virgin areas. ^2^

These applications demonstrate the importance of geophysics in environmental studies, emphasizing its role in understanding environmental anomalies. The objective of this paper is to highlight the crucial role of geophysics in environmental studies and showcase its applications for addressing environmental problems in Indonesia. This will be based on a small bibliometric dataset from the Scopus database. ^3^

## The general applications of geophysics

### Geophysics to solve water-related environmental problems

Geophysics has played a crucial role in addressing various water-related environmental challenges, as demonstrated the improved extraction of hydrologic information from geophysical data through coupled hydrogeophysical inversion, allowing for the monitoring of infiltration and redistribution of water using surface-based electrical conductivity surveys. ^4^ Another work highlighted the integration of geophysical techniques with hydrological investigation for identifying groundwater contamination, emphasizing its frequent use in solving geological and environmental problems related to groundwater quality and exploration. ^2^ Additionally, we found a successful application of geophysical methods in investigating aquifer contamination by various sources such as graveyards, highlighting the versatility of geophysics in addressing environmental problems related to groundwater pollution. ^5^ These examples underscore the pivotal role of geophysics in understanding and mitigating water-related environmental issues, ranging from groundwater contamination to the study of aquifer pollution by diverse sources.

### Geophysics to detect metals from fertile soils and plants

The application of geophysics in detecting metals from fertile soil and plants has been demonstrated in various studies, including those conducted in Indonesia by several researchers from Institut Teknologi Bandung. ^6^ Another instance from North China Plain, researchers have utilized geophysical methods to investigate the accumulation of heavy metals in agricultural soil and products, highlighting the relationship between the application of organic and phosphate fertilizers and the accumulation of cadmium, lead, copper, and zinc in the soil and agricultural products. ^7^ This study underscores the significance of geophysics in understanding the factors contributing to metal accumulation in agricultural areas.

Furthermore, this work has also shed light on the influence of soil heterogeneity on plant development and crop yield, as evaluated using time-series of UAV and ground-based geophysical imagery. ^8^ This research emphasized the integration of multiscale and multitype soil and plant datasets to identify the spatiotemporal co-variance between soil properties and plant development and yield, showcasing the utility of geophysics in understanding the complex interactions between soil and plants.

Moreover, several studies have contributed to the widespread applications of geophysical methods for environmental problems. For example, we found two researchers have investigated the resistance properties of different soil types to heavy metals, particularly in agricultural soils. Their research highlights the role of geophysics in assessing the impact of agricultural practices on soil properties and metal content. It provides valuable insights into the potential effects of long-term fertilization on the accumulation of heavy metals in soil, even in cemetery areas. ^9^^,^^10^

Additionally, geophysics have been used to assess heavy metal accumulation in agricultural soil, understanding soil-plant interactions, evaluating the impact of agricultural practices on metal content in soil and plants, and studying the hyper-accumulation of metal content by certain plants. ^6^

## The applications of geophysics in Indonesia

**Figure 1.**
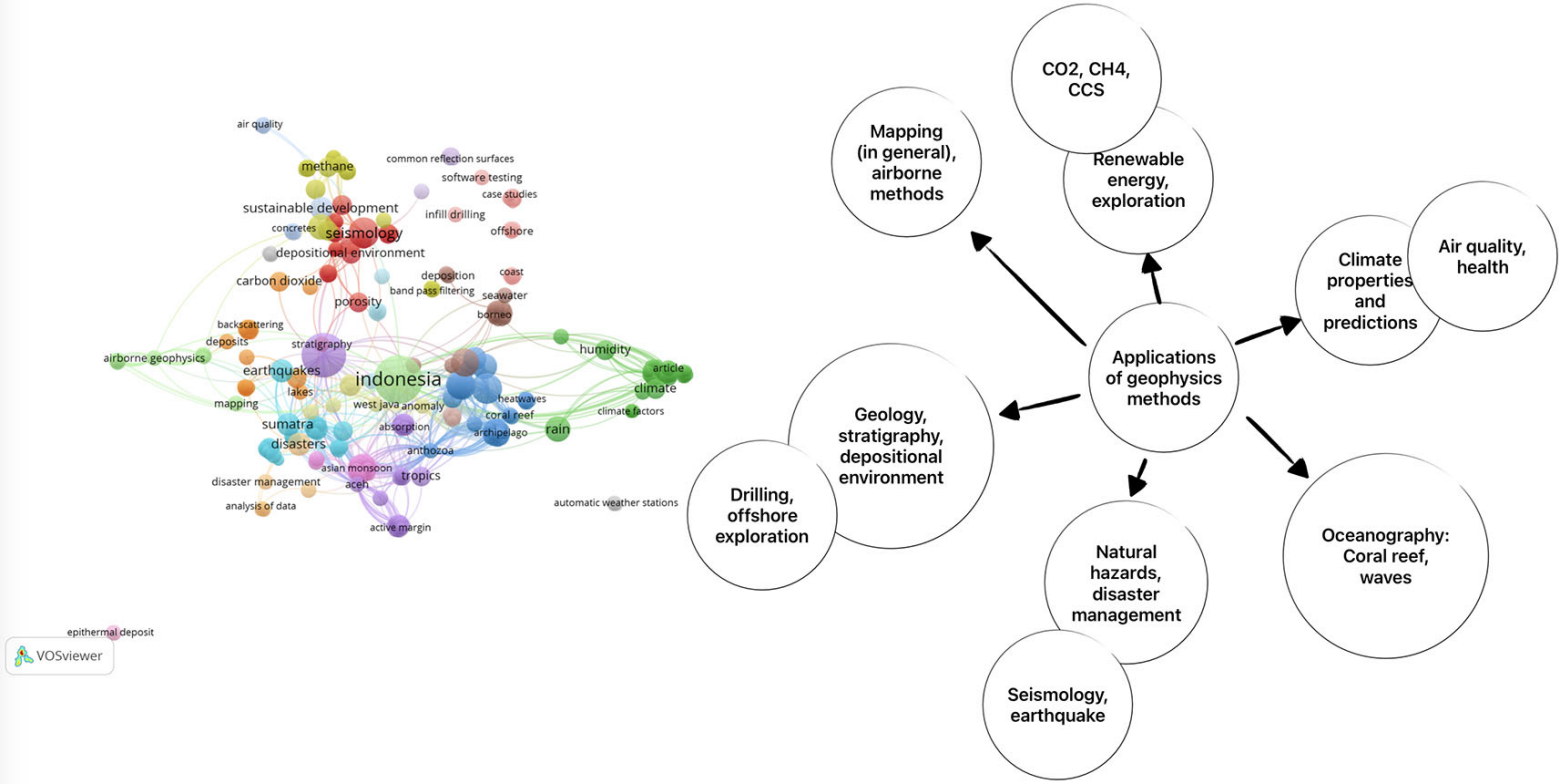
This visualization showcases the breadth and depth of geophysics research conducted in Indonesia.

This section explains the applications of various geophysical methods in Indonesia. To gather scientific articles on this topic, we conducted a search using the Scopus database with the keywords “geophysics” AND “Indonesia”. The search yielded 582 articles. To provide a visual representation of the corpus, we utilized Vosviewer ^11^ and obtained the following result (see Figure 1).

In the diagram below, we present an overview of the applications of geophysics based on a search of the Scopus database. The applications of geophysics in the Scopus database can be categorized into the following areas:

1. **Engineering, constructions, and disaster management**: Geophysics plays a crucial role in engineering and construction projects by providing insights into subsurface conditions, detecting potential hazards, and assessing the structural integrity of buildings and infrastructure. It also aids in the management and mitigation of natural disasters such as earthquakes and landslides.
2. **Climate, meteorology, infectious diseases, public health, agriculture**: Geophysics contributes to understanding climate patterns, meteorological phenomena, and their impact on public health and agriculture. It helps monitor environmental factors, such as air quality, temperature, and rainfall, that influence the spread of infectious diseases and the productivity of agricultural systems.
3. **Oceanography, physical and chemical analyses**: Geophysics is essential for studying the oceans, including their physical and chemical properties. It aids in understanding sea wave behavior, coastal erosion, and the design of offshore structures. Geophysical techniques also contribute to the analysis of water column properties, such as salinity, temperature, and currents, which are crucial for oceanographic research.
4. **Energy exploration, hydrocarbon, geothermal, volcanoes, structural geology, groundwater**: Geophysics plays a vital role in energy exploration, including the identification of hydrocarbon reservoirs, assessment of geothermal resources, and monitoring of volcanic activity. It also aids in studying structural geology and understanding groundwater systems, including their availability and quality.
5. **Seismology, earthquake, earth interior, geodynamics**: Geophysics is fundamental to seismology, the study of earthquakes and the Earth’s interior. It helps detect and monitor seismic activity, analyze earthquake sources, and investigate the dynamics of the Earth’s tectonic plates. Geophysical methods provide valuable insights into the structure and composition of the Earth’s interior.

These categories highlight the broad range of applications of geophysics, demonstrating its significance in various scientific disciplines and practical fields. Geophysics plays a vital role in understanding the Earth’s physical characteristics and addressing environmental challenges.

### Exploring the potential of geophysics in solving environmental challenges

**Figure 2.**
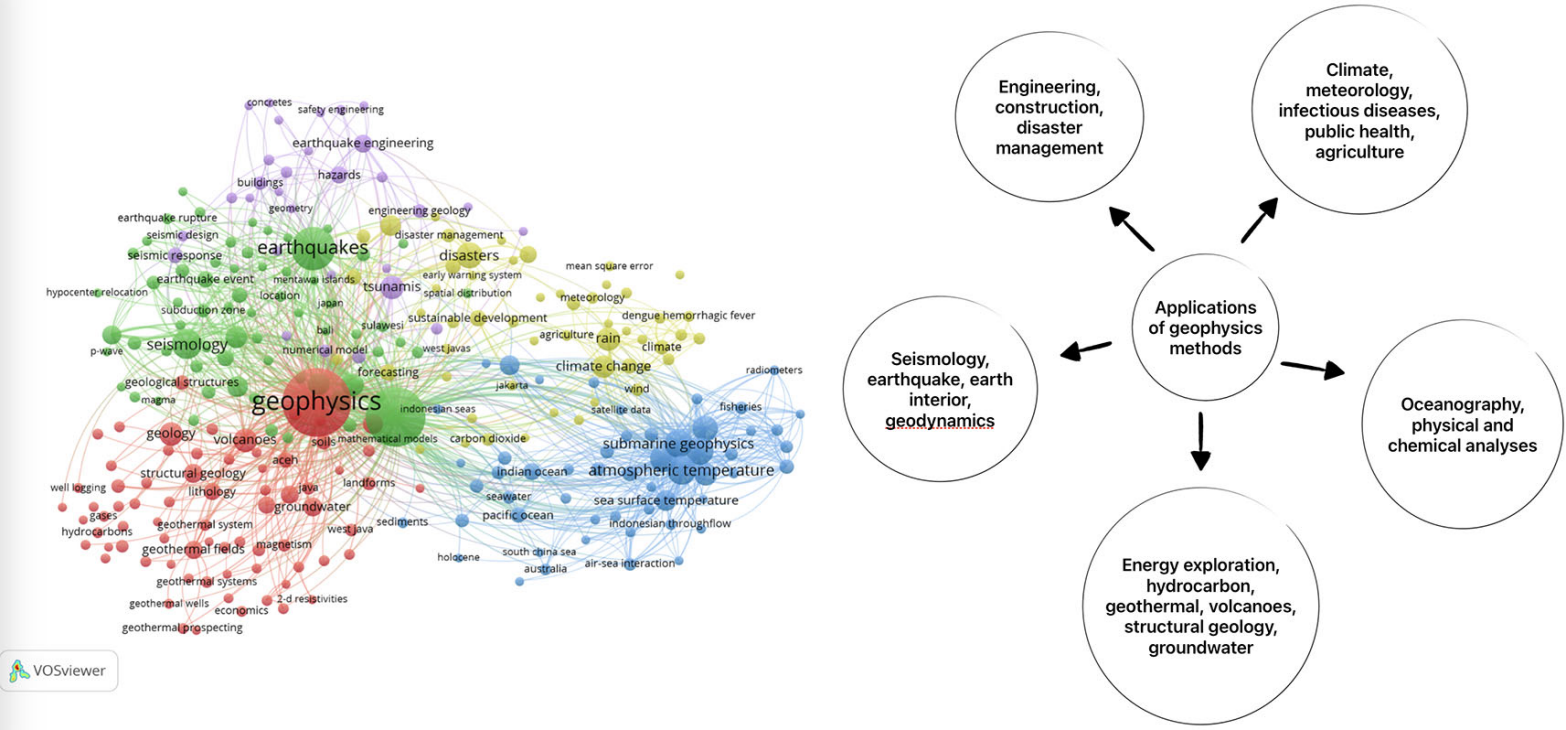
This visualization represents the research articles obtained from the search using the keywords “geophysics,” “Indonesia,” and “environment”.

For a more detailed search focusing on the intersection of geophysics, Indonesia, and the environment, we used the keywords “geophysics” AND “Indonesia” AND “environment”. This search yielded 40 articles, providing a more focused collection of research on these specific topics. The visualization in Figure 2 showcases the breadth and depth of geophysics research conducted in Indonesia, specifically related to environmental studies.

In addition to the applications mentioned in the paper, geophysics is widely used in energy exploration and seismology to identify the structure of the Earth’s interior. By analyzing seismic waves and conducting geophysical surveys, scientists can gain insights into underground formations, including potential energy resources such as oil, gas, and minerals. ^12^ This information is crucial for the exploration and extraction of these resources.

Furthermore, airborne geophysical mapping has proven to be a valuable tool in various applications. The use of aerial platforms equipped with geophysical sensors allows for the rapid and efficient collection of data over large areas. ^13^ This technique has been applied in geological mapping, mineral exploration, and environmental studies. It enables researchers to obtain high-resolution data, which is particularly useful for identifying subsurface structures and mapping geological features.

Geophysics also plays a significant role in oceanography, which is particularly relevant in Indonesia. As an archipelago nation, Indonesia has a vast expanse of ocean, covering approximately 62% of its total area. Geophysics aids researchers in understanding the behavior of sea waves, including their propagation, energy dissipation, and interaction with coastal areas. By studying these processes, scientists can better predict and manage coastal erosion, as well as improve the design and safety of offshore structures. ^14^ Additionally, geophysics contributes to the study of coral reefs in Indonesia by providing insights into their growth patterns, health, and vulnerability to environmental changes.

Surprisingly, geophysics also finds applications in climate properties and predictions related to human health. By monitoring changes in the Earth’s magnetic field and studying atmospheric conditions, scientists can investigate the effects of climate change on human well-being. Geophysical techniques, such as magnetometry and atmospheric measurements, contribute to understanding the links between climate patterns, air quality, and human health outcomes. ^15^ These applications highlight the interdisciplinary nature of geophysics and its potential impact on various aspects of our lives.

### Limitations of geophysical approaches at a practical level in Indonesia

Geophysics is a valuable tool for environmental investigations and remediation. However, it is important to be aware of the limitations and use it in conjunction with other techniques.

Geophysical methods allow for the quick and efficient mapping of large land areas, which is crucial for identifying and characterizing potential contamination sites. ^16^ Despite being costly, they provide significant advantages by efficiently assessing contamination extent and identifying potential sources. These non-invasive methods minimize disturbance to the environment and save time and resources.

Geophysics offers a non-destructive approach to investigating the Earth’s physical characteristics. Electrical resistivity tomography (ERT) can map groundwater contamination plumes by imaging subsurface resistivity distribution. Ground penetrating radar (GPR) detects buried objects and structures, aiding in environmental site assessments and remediation. Electromagnetic induction (EMI) maps salt distribution in soil, indicating potential groundwater contamination areas. ^17^

It’s important to note that geophysical methods may not detect all types of contamination, such as volatile organic compounds (VOCs), and interpreting data in complex geological settings can be challenging. ^18^ Nonetheless, they provide valuable insights into subsurface geology, helping assess groundwater contamination potential and guide well and sampling point placement.

However, despite its advantages, applying geophysics in dense urban areas can be challenging due to several factors. Here are some of the key challenges:

• **Physical obstructions**: Urban environments are characterized by a dense network of infrastructure, including buildings, roads, bridges, and underground utilities. These structures can obstruct access to the subsurface, making it difficult to deploy geophysical equipment and collect data.
• **Cultural noise**: Urban areas generate significant cultural noise, including traffic vibrations, electromagnetic interference from electronic devices, and human activity. This noise can mask the subtle geophysical signals that geophysicists are trying to measure.
• **Limited workspace**: Urban areas often have limited open space, making it challenging to set up and operate geophysical equipment. This can be particularly problematic for methods that require large arrays of sensors or long survey lines.
• **Permits and regulations**: Urban environments are often subject to strict regulations and permit requirements, which can add time and complexity to geophysical surveys.
• **Safety concerns**: Working in urban areas can pose safety risks, including exposure to traffic, hazardous materials, and confined spaces. Geophysicists must carefully plan and execute their surveys to minimize these risks.

Despite these challenges, geophysics can still be a valuable tool for urban applications. Geophysicists have developed a variety of techniques that can be adapted to urban environments, and new technologies are being developed all the time.

## Remarks

The document discusses the hidden potential of geophysics for the environment, with a focus on Indonesia. It highlights the role of geophysics in understanding the Earth’s physical characteristics and its applications in addressing environmental challenges. The paper explores the use of geophysics in solving water-related environmental problems, detecting metals in fertile soils and plants, and its relevance in the Indonesian context. It also provides an overview of the broad applications of geophysics in the world, including engineering, climate, oceanography, energy exploration, and seismology. The limitations and challenges of applying geophysics in urban areas are also discussed.

Below are some important remarks that should be noted and developed by future earth scientists, particularly Indonesian earth scientists. These remarks provide directions for future research and highlight the potential for further development and collaboration in the field of geophysics for environmental studies, particularly in the Indonesian context.

1. **Integration of geophysics and environmental studies**: The document emphasizes the importance of integrating geophysics with environmental studies to address environmental challenges effectively. Future researchers should continue exploring the interdisciplinary nature of geophysics and its applications in various environmental contexts.
2. **Water-related environmental problems**: Geophysics plays a crucial role in addressing water-related environmental problems, such as groundwater contamination and infiltration monitoring. Researchers should further investigate the potential of geophysics in assessing water quality and exploring innovative techniques for sustainable water management.
3. **Metal detection in soils and plants**: The use of geophysics for detecting metals in fertile soils and plants provides valuable insights into agricultural practices and potential health risks. Further research is needed to expand this application and explore its implications for food security and environmental sustainability.
4. **Urban applications of geophysics**: Applying geophysics in dense urban areas presents unique challenges due to physical obstructions, cultural noise, limited workspace, permits, and safety concerns. Researchers should focus on developing techniques and methodologies specifically tailored for urban environments to overcome these challenges and unlock the full potential of geophysics in urban applications.
5. **Indonesian context**: Geophysics plays a significant role in Indonesia due to its diverse geological and environmental characteristics. Future research should concentrate on understanding the specific environmental challenges faced by Indonesia and developing innovative geophysical approaches to address these issues effectively.
6. **Advancements in technology**: Continued advancements in geophysical technology, such as aerial platforms, improved sensors, and data analysis techniques, offer promising opportunities for expanding the applications of geophysics. Researchers should stay updated with emerging technologies and incorporate them into their studies to enhance the accuracy and efficiency of geophysical investigations.

## Data Availability

No data are associated with this article. Figshare: Extended data for ‘Geophysics for the environment in Indonesia’, https://doi.org/10.6084/m9.figshare.24759996.v2. ^3^ Data are available under the terms of the Creative Commons Zero “No rights reserved” data waiver (CC0 1.0 Public domain dedication).
