# Geophysics for the environment in Indonesia

**DOI:** 10.12688/f1000research.145869.2

**Published:** 2026-05-04

**Authors:** Achmad Darul, Dasapta Erwin Irawan, Eleonora Agustine

**Affiliations:** 1 Departement of Geology, Faculty of Industrial Engineering, Institut Teknologi Sumatera, Way Hui, South Lampung, Indonesia; 2 Applied Geology Research Group, Faculty of Earth Sciences and Technology, Institut Teknologi Bandung, Bandung, West Java, Indonesia; 3 Faculty of Geology, Universitas Padjadjaran, Bandung, West Java, Indonesia

**Keywords:** geophysics, environmental problems, urban areas, urban hydrogeology, subsurface analyses, geology, aquifers, groundwater

## Abstract

Environmental geophysics holds significant but underutilized potential for tackling Indonesia’s diverse environmental challenges by supporting investigations of groundwater contamination, water infiltration, and the accumulation of metals in soils and crops—issues crucial for agriculture, public health, and sustainable land management. Emerging technologies such as UAV-assisted imaging, airborne surveys, and oceanographic geophysical observations further demonstrate the flexibility of modern geophysics in studying coastal processes, wave behaviour, and coral reef conditions. However, its application—especially in urban Indonesia—remains limited due to physical obstructions, cultural noise, restricted workspace, regulatory hurdles, and safety concerns, while the absence of geophysical test sites (GTS) and a shortage of skilled practitioners constrain methodological advancement and training. Based on bibliometric analysis of Scopus-indexed publications, this study shows that integration between geophysics and environmental science is growing but still insufficient. Strengthening environmental geophysics in Indonesia requires developing dedicated training and calibration facilities, fostering interdisciplinary collaboration, and adopting technological innovations tailored to dense urban and complex geological settings so that geophysics can play a more effective role in environmental monitoring, resource sustainability, and resilience to ecological and urban challenges.

## Introduction

Geophysics plays a crucial role in environmental studies by providing valuable insights into the Earth’s physical characteristics and the living environment of mankind. Environmental geophysics, also known as near-surface geophysics, integrates environmental science and geophysics to study the relationship between geophysical fields and the Earth’s physical characteristics, including natural and artificial environments. ^1^ ^,^ ^2^ Geophysical techniques are pivotal tools for detecting and studying various environmental problems caused by drinking and wastewater, as well as delineating contaminated, partly affected, and virgin areas. ^3^ ^,^ ^4^

Environmental geophysics, formally emerging as a distinct interdisciplinary field in the late 1980s, integrates geophysical principles with environmental science to detect, monitor, and solve complex ecological issues. Globally, the application of geophysical methods has expanded rapidly due to their distinct advantages: they are cost-effective, fast, highly accurate, non-destructive, and produce no secondary pollution. These methods are now widely deployed across various domains, including air quality monitoring, marine oil spill detection, radioactive pollutant tracking, and solid waste management. For instance, recent studies have demonstrated the critical role of electrical resistivity surveys in mapping dumpsite leachate plumes that contaminate groundwater, thereby mitigating severe structural and public health risks. Despite these global advancements, the development and application of environmental geophysics in certain developing regions remain slow. ^5^ ^–^ ^7^

These applications demonstrate the importance of geophysics in environmental studies, emphasizing its role in understanding environmental anomalies. The objective of this paper is to highlight the crucial role of geophysics in environmental studies and showcase its applications for addressing environmental problems in Indonesia. This will be based on a small bibliometric dataset from the Scopus database.

## The general applications of geophysics

### Geophysics to solve water-related environmental problems

By utilizing coupled hydrogeophysical inversion, researchers can better extract hydrologic information to monitor water infiltration and redistribution through surface-based electrical conductivity. Additionally, combining geophysical techniques with hydrological studies allows for the detection of groundwater pollutants, a common practice in modern geological and environmental efforts to manage groundwater quality. ^8^ ^,^ ^9^

Coupled hydrogeophysical inversion improves hydrologic information, enabling better monitoring of water infiltration and movement in the subsurface. Combined geophysical–hydrological methods also support the detection of groundwater pollutants and aquifer contamination, highlighting the importance of geophysics in managing groundwater quality and related environmental challenges. ^10^ ^,^ ^11^

### Geophysics to detect metals from fertile soils and plants

The use of geophysics to detect metals in fertile soils and plants has been demonstrated across multiple studies, including work conducted in Indonesia. One study applied geophysical methods to evaluate the accumulation of heavy metals in agricultural soils and crop products in the North China Plain, revealing how the application of organic and phosphate fertilizers can influence the buildup of cadmium, lead, copper, and zinc. This highlights the value of geophysical approaches for understanding the factors that drive metal accumulation in agricultural settings. ^10^ ^–^ ^12^

The application of geophysics to detect metals in fertile soils and plants has been demonstrated across numerous studies, including work conducted in Indonesia. One study applied geophysical methods to assess the accumulation of heavy metals in agricultural soils and crop products in the North China Plain, showing how the use of organic and phosphate fertilizers influences the buildup of cadmium, lead, copper, and zinc. These findings highlight the importance of geophysical approaches for understanding the factors that contribute to metal accumulation in agricultural environments. ^13^ ^–^ ^15^

This work has also shed light on the influence of soil heterogeneity on plant development and crop yield, as evaluated using time-series of UAV and ground-based geophysical imagery. This research emphasized the integration of multiscale and multitype soil and plant datasets to identify the spatiotemporal co-variance between soil properties and plant development and yield, showcasing the utility of geophysics in understanding the complex interactions between soil and plants. ^16^

Several studies have demonstrated the broad usefulness of geophysical methods for addressing environmental challenges. Research on various soil types has shown how geophysical techniques can reveal differences in their resistance to heavy metals, particularly in agricultural settings. These findings underscore the value of geophysics in evaluating how farming practices influence soil characteristics and metal concentrations. Such work also provides important insights into how long-term fertilizer use can lead to the gradual accumulation of heavy metals in the soil, including in locations with unique land uses such as cemeteries. ^17^

### Airborne Geophysics for detection of geological features

Airborne geophysical mapping has also become a highly effective tool across many fields. Using aerial platforms equipped with specialized sensors, researchers can rapidly collect data over wide areas with high efficiency. This approach is applied in geological mapping, mineral exploration, and various environmental investigations. The ability to generate high-resolution datasets makes airborne methods particularly valuable for identifying subsurface structures and delineating geological features. ^18^

### Geophysics for oceanography applications

Geophysics also plays an important role in oceanography, a field that is especially relevant for Indonesia as an archipelago nation with more than half of its territory covered by ocean. Through geophysical observations, researchers can study the behaviour of sea waves, including how they propagate, lose energy, and interact with coastal environments. Understanding these processes helps improve predictions of coastal erosion and supports the design and safety of offshore structures. Geophysics is also widely used to study coral reefs in Indonesia, offering insights into their growth patterns, overall health, and vulnerability to environmental change. ^19^

### Geophysics for climate observation

Geophysics also contributes to studies of climate processes and their connections to human health. By observing variations in the Earth’s magnetic field and examining atmospheric conditions, scientists can explore how climate-related changes influence human well-being. Techniques such as magnetometry and atmospheric monitoring help reveal relationships between climate patterns, air quality, and health outcomes. These applications highlight the interdisciplinary nature of geophysics and its potential impact across many aspects of daily life. ^20^ ^,^ ^21^

## The applications of geophysics in Indonesia

**Figure 1.**
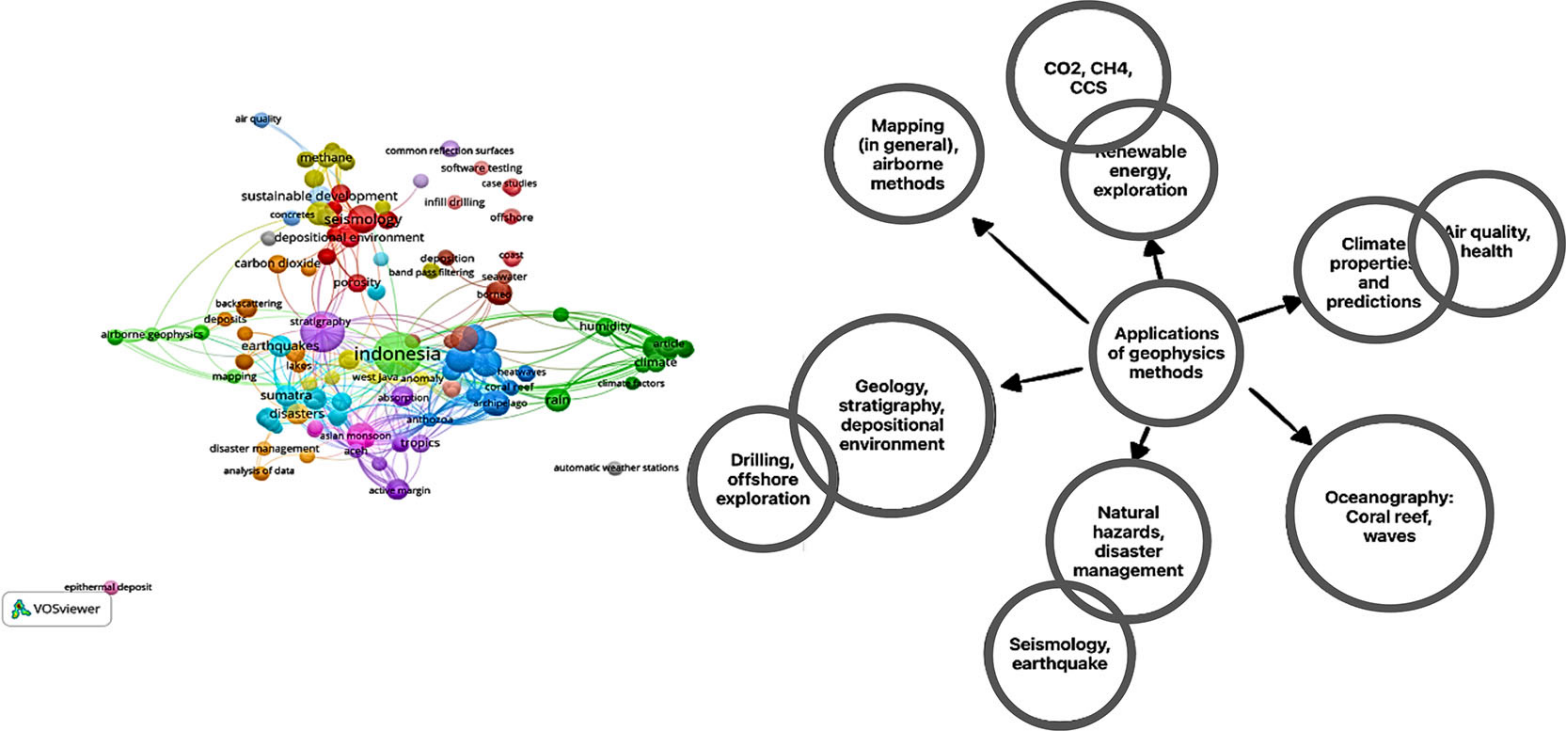
This visualization showcases the breadth and depth of geophysics research conducted in Indonesia.

This section explains the applications of various geophysical methods in Indonesia. To gather scientific articles on this topic, we conducted a search using the Scopus database with the keywords “geophysics” AND “Indonesia”. The search yielded 582 articles. To provide a visual representation of the corpus, we utilized Vosviewer ^22^ and obtained the following result (see Figure 1).

In the diagram below, we present an overview of the applications of geophysics based on a search of the Scopus database. The applications of geophysics in the Scopus database can be categorized into the following areas:

1. **Engineering, Constructions, and Disaster Management**: Geophysics plays a crucial role in engineering and construction projects by providing insights into subsurface conditions, detecting potential hazards, and assessing the structural integrity of buildings and infrastructure. It also aids in the management and mitigation of natural disasters such as earthquakes and landslides.
2. **Climate, Meteorology, Infectious Diseases, Public Health, Agriculture**: Geophysics contributes to understanding climate patterns, meteorological phenomena, and their impact on public health and agriculture. It helps monitor environmental factors, such as air quality, temperature, and rainfall, that influence the spread of infectious diseases and the productivity of agricultural systems.
3. **Oceanography, Physical and Chemical Analyses**: Geophysics is essential for studying the oceans, including their physical and chemical properties. It aids in understanding sea wave behavior, coastal erosion, and the design of offshore structures. Geophysical techniques also contribute to the analysis of water column properties, such as salinity, temperature, and currents, which are crucial for oceanographic research.
4. **Energy Exploration, Hydrocarbon, Geothermal, Volcanoes, Structural Geology, Groundwater**: Geophysics plays a vital role in energy exploration, including the identification of hydrocarbon reservoirs, assessment of geothermal resources, and monitoring of volcanic activity. It also aids in studying structural geology and understanding groundwater systems, including their availability and quality.
5. **Seismology, Earthquake, Earth Interior, Geodynamics**: Geophysics is fundamental to seismology, the study of earthquakes and the Earth’s interior. It helps detect and monitor seismic activity, analyse earthquake sources, and investigate the dynamics of the Earth’s tectonic plates. Geophysical methods provide valuable insights into the structure and composition of the Earth’s interior.

These categories highlight the broad range of applications of geophysics, demonstrating its significance in various scientific disciplines and practical fields. Geophysics plays a vital role in understanding the Earth’s physical characteristics and addressing environmental challenges.

**Figure 2.**
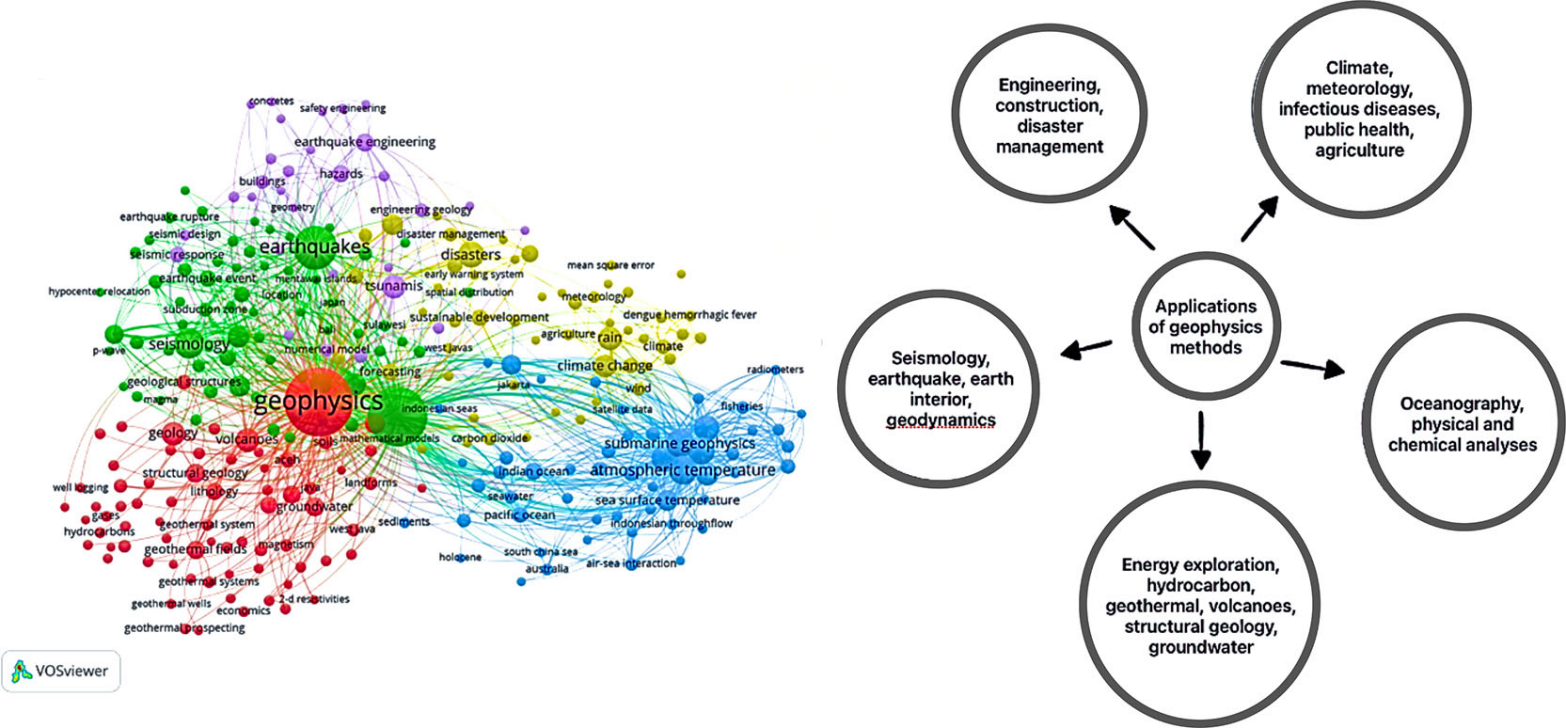
This visualization represents the research articles obtained from the search using the keywords “geophysics,” “Indonesia,” and “environment”.

For a more detailed search focusing on the intersection of geophysics, Indonesia, and the environment, we used the keywords “geophysics” AND “Indonesia” AND “environment”. This search yielded 40 articles, providing a more focused collection of research on these specific topics. The visualization below ( Figure 2) showcases the breadth and depth of geophysics research conducted in Indonesia, specifically related to environmental studies.

In addition to the applications discussed earlier, geophysics is extensively used in energy exploration and seismology to reveal the structure of the Earth’s interior. By analysing seismic waves and conducting subsurface surveys, scientists can better understand underground formations, including potential resources such as oil, gas, and minerals. These insights are essential for guiding exploration activities and supporting resource extraction. ^23^

## Limitations of geophysical approaches in practical level in Indonesia

Although geophysical methods are widely used in environmental investigations, their application in Indonesia faces several practical limitations. These challenges are shaped by local geological conditions, urban complexity, regulatory constraints, and capacity gaps within the professional community. The following subsections summarize these limitations. ^24^

### Technical and environmental constraints

Complex geological conditions. Interpreting geophysical data in heterogeneous or layered subsurface environments remains difficult, particularly where soil conductivity, moisture, or lithological contrasts vary significantly. These conditions can obscure anomalies or lead to ambiguous interpretations. Similar challenges have been documented globally, but are often mitigated through integrated surveys or the use of multi-method inversion workflows.

Limited suitability for certain contaminants. Some contaminants—especially volatile organic compounds (VOCs)—produce weak or non-distinct geophysical signatures, making them difficult to detect using methods such as Electrical resistivity tomography (ERT), Ground penetrating radar (GPR), and Electromagnetic induction (EMI). ^25^ It’s important to note that geophysical methods may not detect all types of contamination. In many countries, this limitation is addressed through combined geophysical–geochemical investigations or by coupling geophysics with direct sampling. ^26^

### Urban-related constraints

Urban environments in Indonesia present a distinct set of challenges for geophysical surveys. Dense infrastructure, high levels of cultural noise, and limited available space often restrict instrument deployment and reduce data quality. These constraints are not unique to Indonesia but tend to be more pronounced in rapidly growing urban areas, making near-surface investigations particularly difficult and requiring careful adaptation of survey design.

• Physical obstructions. Dense infrastructure (roads, utilities, buildings) restricts sensor deployment and complicates data acquisition.
• Cultural noise. Urban electromagnetic noise, traffic vibrations, and anthropogenic interference often mask geophysical signals, reducing data quality. In other regions, noise-reduction strategies—such as time-window filtering, shielded antennas, or nighttime acquisition—are commonly adopted.
• Limited workspace. Survey lines, sensor arrays, and transmitter–receiver setups often require space that urban Indonesia does not easily provided.
• Permitting and regulatory difficulties. Survey activities in public or sensitive spaces require multiple layers of permission, extending project timelines.
• Safety concerns. Fieldwork in traffic-dense or hazardous urban zones demands additional safety planning and personnel protection measures.

### Institutional and capacity limitations

Insufficient interdisciplinary coordination. Adoption of environmental geophysics is slowed by weak collaboration between geophysicists, environmental managers, and policymakers, as well as a lack of dedicated applied-research funding.

Shortage of trained experts and absence of Geophysical Test Sites (GTS). The lack of controlled training and calibration facilities creates a persistent skills gap. Students and early-career practitioners often learn theory without opportunities to practice on real or simulated subsurface targets. As a result, methodological mastery—especially in inversion, processing, and validation—remains. ^27^

In contrast, many geophysics programs worldwide rely heavily on GTS to strengthen field competence, calibrate new instruments, and validate methods before deployment in urban or environmentally sensitive settings. Such facilities have been shown to significantly improve the reliability of near-surface investigations and the readiness of geophysical workers to address complex subsurface problems. ^28^

Globally, many of these challenges have been addressed through methodological integration, multi-sensor approaches, improved inversion algorithms, and the establishment of GTS-based training frameworks. Incorporating similar strategies within Indonesia may help reduce uncertainties and strengthen the effectiveness of environmental geophysics across diverse settings.

## Conclusions

This paper has outlined the specific opportunities and challenges associated with advancing environmental geophysics within the Indonesian context. While many geophysical approaches for environmental assessment are well-established and widely implemented in other parts of the world, their adoption in Indonesia remains comparatively limited. This gap is shaped not by the global state of the discipline but by local constraints, including urban obstructions, operational noise, limited field space, regulatory complexity, and the shortage of dedicated training facilities such as Geophysical Test Sites (GTS).

To strengthen the role of geophysics in addressing Indonesia’s environmental challenges—ranging from groundwater contamination and landfill management to soil metal accumulation in agricultural regions—capacity building is essential. The establishment of GTS can help bridge the gap between theory and practical competence, supporting improved field training, equipment calibration, and method validation in diverse soil conditions. Enhanced integration between geophysics, environmental science, and policy frameworks is also critical, particularly for issues such as groundwater protection and sustainable land management.

Indonesia’s unique geological, environmental, and urban conditions require methodological adaptations rather than the wholesale adoption of practices used elsewhere. Advances in sensor technologies, UAV-based platforms, and improved subsurface modelling tools provide pathways for tailoring geophysical approaches to these challenges. Leveraging these innovations will support stronger environmental monitoring, public-health protection, and the development of resilient infrastructure across Indonesia.

## Data availability

### Underlying data

No data are associated with this article.

### Extended data

Figshare: Extended data for ‘Geophysics for the environment in Indonesia’, https://doi.org/10.6084/m9.figshare.24759996.v2. ^3^

Data are available under the terms of the Creative Commons Zero “No rights reserved” data waiver (CC0 1.0 Public domain dedication).

## Author contributions

All authors contributed equally to the conception, design, execution, analysis, and interpretation of the work, and drafting and revising of the manuscript. All authors approve the final version of the manuscript for submission.

## AI declaration

Artificial intelligence tools were used in the preparation of this manuscript strictly for language-related assistance. Microsoft Copilot was employed to translate parts of the manuscript from Indonesian to English using the prompt: “translate this text to English using straight-forward academic English, but don’t lose the narrative.” Microsoft Copilot was also used for proofreading and language polishing using the prompt: “conduct a comprehensive proofreading and language smoothing for easier understanding.”

All scientific interpretations, methodological descriptions, data analyses, conclusions, and overall intellectual content were developed entirely by the authors. The authors reviewed and verified all AI-assisted outputs to ensure accuracy and integrity. No AI tool was used for generating analysis, results, or original scientific content.
