# Supplementary material for: Geophysics for the environment in Indonesia
Source: F1000Res. 2026 May 4;13:131. Originally published 2024 Feb 22. [Version 2] doi: 10.12688/f1000research.145869.2 (PMC13191308; doi:10.12688/f1000research.145869.2)
Supplement: Peer review history

# Reviewer report: Reviewer response for version 2

**DOI:** 10.5256/f1000research.197616.r482375

**Published:** 2026-05-20

**Authors:** Sribas Kanji

**Affiliations:** 1 Jadavpur University, Kolkata, West Bengal, India

**Comments:**

1. **Abstract**
  1. Split the long sentences to improve clarity and readability.
  2. The abstract is more likely to be written using large language models or AI. Unstructured abstract required revision, rewrite the abstract following the sequence – Research purpose, gap and question, novelty, research findings, and overall statement.
2. ** Introduction**
  1. This paper does not sufficiently or convincingly justify why this review is necessary. While it may provide general background information, it lacks a clear identification of specific gaps, inconsistencies, or unresolved issues in the existing literature that warrant a dedicated review. The argument for necessity appears broad and descriptive rather than critical and focused. To strengthen this section, the authors should explicitly articulate what is missing in current studies, how this review addresses those shortcomings, and what new insights or synthesis it aims to contribute beyond existing reviews.
  2. The objectives of the study are not clearly or precisely defined, which limits the overall focus and direction of the paper. The current formulation appears too broad and lacks specific, measurable, and research-oriented questions. A well-defined objective should explicitly state what the study aims to investigate, under what conditions, and with what expected outcomes.
  3. Furthermore, the absence of clearly articulated objectives makes it difficult to assess whether the methodology, model selection, and results are appropriately aligned with the intended scope. The objectives should be refined to be concise, specific, and directly linked to the research gap and novelty of the study, ensuring a coherent flow between the problem statement, analysis, and conclusions.
3. **Structure**
  1. The manuscript suffers from a poor overall structure, which significantly affects its clarity and readability. The flow of information between sections is not well organized, making it difficult for readers to follow the research objectives, methodology, results, and conclusions in a logical sequence. Key components such as the background, research gap, methodology, and discussion are either insufficiently developed or not clearly delineated. Additionally, transitions between sections are weak, leading to fragmentation of ideas. A more coherent and systematically organized structure following a standard scientific format with clearly defined sections and logical progression is essential to improve the comprehensibility and academic quality of the manuscript.
  2. There is a lack of *thematic synthesis and critical comparison*, making it difficult to identify key trends, methodological differences, and unresolved debates across studies.
  3. The absence of well-defined *subsections with informative headings* reduces the structural clarity of the review, leading to a fragmented presentation of ideas. This weak organization hinders readability and obscures the logical progression of arguments.
  4. The lack of *visual synthesis tools* such as comparative tables, conceptual frameworks, or flowcharts misses an opportunity to effectively illustrate relationships, similarities, and differences among studies. Without these elements, the review remains largely narrative and does not fully support critical insight or knowledge integration.
4. ***Discussion and Future Perspectives***
  1. The *Discussion *section appears to be conceptually structured but lacks sufficient depth and critical rigor. While it claims to address strengths, weaknesses, research gaps, and future directions, these components need to be more explicitly articulated and supported by evidence from the results and existing literature.
  2. The *future directions* lack specificity and actionable insight. Rather than broad suggestions, this section should propose concrete pathways.
5. **Conclusion**
  1. Revise the conclusion after a thorough revision of the raised issues.

Is the topic of the opinion article discussed accurately in the context of the current literature?

No

Are arguments sufficiently supported by evidence from the published literature?

Partly

Are all factual statements correct and adequately supported by citations?

Partly

Are the conclusions drawn balanced and justified on the basis of the presented arguments?

No

Reviewer Expertise:

Geography, Remote Sensing, Hydrology

I confirm that I have read this submission and believe that I have an appropriate level of expertise to confirm that it is of an acceptable scientific standard, however I have significant reservations, as outlined above.

# Reviewer report: Reviewer response for version 2

**DOI:** 10.5256/f1000research.197616.r481361

**Published:** 2026-05-14

**Authors:** Pradeep Naik

**Affiliations:** 1 Centre for Hydrological Sciences and Communication, Bhubaneswar, Odisha, India; 2 Ministry of Jal Shakti, Govt. of India, Central Ground Water Board, North Central Chhattisgarh Region, Raipur, Chattisghar, India

It may be indexed.

Is the topic of the opinion article discussed accurately in the context of the current literature?

Partly

Are arguments sufficiently supported by evidence from the published literature?

Yes

Are all factual statements correct and adequately supported by citations?

Yes

Are the conclusions drawn balanced and justified on the basis of the presented arguments?

Partly

Reviewer Expertise:

NA

I confirm that I have read this submission and believe that I have an appropriate level of expertise to confirm that it is of an acceptable scientific standard.

# Reviewer report: Reviewer response for version 1

**DOI:** 10.5256/f1000research.159883.r263222

**Published:** 2024-05-07

**Authors:** Pradeep Naik, Dr. Subhash Chandra Singh

**Affiliations:** 1 Centre for Hydrological Sciences and Communication, Bhubaneswar, Odisha, India; 2 Ministry of Jal Shakti, Govt. of India, Central Ground Water Board, North Central Chhattisgarh Region, Raipur, Chattisghar, India; 3 Central Ground Water Board, Central Ground Water Board, Bhopal, Madhya Pradesh, India

This paper describes the virtues of geophysics with a focus on potential environmental applications in Indonesia. The paper is written well, but is too repetitive, meaning the same matters are being conveyed in different ways.

 Few specific observations are as follows:

1. Abstract: Too long; may be substantially reduced. Only one paragraph.
2. Keywords: Words mentioned in the title may be avoided.
3. Introduction: May include the next section, i.e. the general applications, as well.  
4. The general applications of geophysics: May be merged into the Intro. The first sentence is too long. Does not match with the sentences authors have generally framed. May be broken into pieces for clarity. The first para has tense problem. Please use present tense as used elsewhere for coherence purposes.
5. The applications of geophysics in Indonesia: The colour shades and sizes in Fig. 1 may be explained in the legend. The captions ‘geophysics methods’ may be stated as ‘geophysical methods'. Abbreviations may be expanded in the legend.
6. Exploring the potential of geophysics in solving environmental challenges: Are these 40 articles included the 582 articles of the previous section? Same observations in Fig. 2 as well.
7. Limitations: Only limitations may be described in this section. Solutions, if any, to overcome these challenges may be described, if authors desire to. In fact, how these challenges were overcome by the workers in different parts of the world would really be an interesting thing to read about. The advantages of geophysical techniques mentioned in the initial portion of this section may be included in the Intro or the previous sections.
8. Remarks: This section may be termed as ‘Conclusions’ or ‘Concluding Remarks’. But it again repeats the same matters stated in the Intro. Are the listed issues not addressed presently by the geophysical workers world-wide? Or, the authors limit their remarks to Indonesia only – may be clarified.

Is the topic of the opinion article discussed accurately in the context of the current literature?

Partly

Are arguments sufficiently supported by evidence from the published literature?

Yes

Are all factual statements correct and adequately supported by citations?

Yes

Are the conclusions drawn balanced and justified on the basis of the presented arguments?

Partly

Reviewer Expertise:

Hydrogeology

We confirm that we have read this submission and believe that we have an appropriate level of expertise to confirm that it is of an acceptable scientific standard, however we have significant reservations, as outlined above.

# Response

**Published:** 2026-03-10

**Authors:** Dasapta Erwin Irawan

**Affiliations:** Applied Geology Research Group, Institut Teknologi Bandung, Bandung, West Java, Indonesia

We thank the reviewer for these helpful and constructive comments.

 We agree that the introduction, especially the general part, can be further enriched. In the revision we will broaden the overview of environmental and engineering applications of geophysics and its role in education and professional practice, and we will consult the suggested papers where they are relevant.

 For Figures 1 and 2, we acknowledge their current limitations. We will regenerate the VOSviewer maps at the maximum resolution available and improve the captions and legends so that colours, node sizes, and abbreviations are explained more clearly, within the constraints of the software.

 In the section “Limitations of geophysical approaches at a practical level in Indonesia”, we will expand the discussion on funding constraints, the limited number of experts, and the absence of dedicated geophysical test sites (GTS) for training young geophysicists, drawing on the suggested literature where appropriate.

 We will also sharpen and shorten the conclusion so that it is more focused, accurate, and less repetitive, and we will carefully revise the reference list to make the formatting consistent with the journal’s style.

# Reviewer report: Reviewer response for version 1

**DOI:** 10.5256/f1000research.159883.r263217

**Published:** 2024-05-07

**Authors:** Joseph Omeiza Alao

**Affiliations:** 1 Air Force Institute of Technology, Kaduna, Nigeria

Dear Editor,

 I have completed reviewing the manuscript. I am sorry for doing this later.

 The paper by Darul et al., titled “ *Geophysics for the environment in Indonesia*” contains two basic approaches to modelling surface and groundwater.  The paper is an OPINION ARTICLE that provides an important topic bordering society. However, the work requires more generation information in the introductory part, especially the application of Geophysics in engineering and also discusses the importance of geophysics as a professional course and education to widen the global scientific interest. So, the author should strive to improve on that. In addition, the work is good for publication as an opinion article. 

1. **Introduction:** the introduction is **not** **rich** enough and needs more relevant information, especially the general part. The author can read the following articles to improve the manuscript quality if he/she finds them relevant [https://doi.org/10.1007/s11600-023-01096-3; https://doi.org/10.1007/s10064-023-03076-9; https://doi.org/10.1007/s40328-022-00398-0 ; https://doi.org/10.1016/j.scitotenv.2023.165469; DOI: 10.26855/oajrcap.2021.12.001]. Please, note that you are not in any way compelled or forced to cite these articles.
2. **Figure:** figures 1 and 2 are blunt, please provide a better figure with high resolution.
3. Under “ ***Limitations of geophysical approaches at a practical level in Indonesia***” provide more information on funding, inadequate experts, and lack of poor geophysical experimental sites like Geophysical Test Site (GTS) to train young geophysicists in the country. You can obtain information in the suggested articles above.
4. **Conclusion**: please, present a more accurate, sharp, and pointed conclusion.
5. **Refinances**: the reference styles are good but not consistent.

Best wishes

 Thank.

Is the topic of the opinion article discussed accurately in the context of the current literature?

Partly

Are arguments sufficiently supported by evidence from the published literature?

Yes

Are all factual statements correct and adequately supported by citations?

Yes

Are the conclusions drawn balanced and justified on the basis of the presented arguments?

Partly

Reviewer Expertise:

Applied GeophysicsGephysics specialize in environmental and geotechnical geophysics

I confirm that I have read this submission and believe that I have an appropriate level of expertise to confirm that it is of an acceptable scientific standard, however I have significant reservations, as outlined above.

## References

[1] : Geophysical test site in teaching, researching, field-based exercises and solving real-life work situations: a case study. *Acta Geodaetica et Geophysica* .2022; 10.1007/s40328-022-00398-0 10.1007/s40328-022-00398-0

[2] : Effects of dumpsite leachate plumes on surface and groundwater and the possible public health risks. *Sci Total Environ* .2023;897: 10.1016/j.scitotenv.2023.165469 165469 10.1016/j.scitotenv.2023.165469 37442480

[3] : Application of geophysical methods in environmental monitoring. *OAJRC Applied Physics* .2022;3(1) : 10.26855/oajrcap.2021.12.001 1-4 10.26855/oajrcap.2021.12.001

# Response

**Published:** 2026-03-10

**Authors:** Dasapta Erwin Irawan

**Affiliations:** Applied Geology Research Group, Institut Teknologi Bandung, Bandung, West Java, Indonesia

We thank the reviewer for the constructive and encouraging comments on our opinion article.

 1. We agree that the introduction, especially the general part, needs to be enriched. We will expand the discussion on broader environmental and engineering applications of geophysics, as well as its role in education and professional training, and will consult the suggested articles where relevant.

 2. We will replace Figures 1 and 2 with higher‑resolution images (if possible) and improve the caption to better resemble the image, so that the bibliometric visualizations are clearer.

 3. In the section “Limitations of geophysical approaches at a practical level in Indonesia”, we will add a more explicit discussion on funding limitations, the shortage of experts, and the absence of dedicated geophysical test sites (GTS) for training young geophysicists, using the suggested references where appropriate.
